# Characterization of Signal Quality Monitoring Techniques for Multipath Detection in GNSS Applications

**DOI:** 10.3390/s17071579

**Published:** 2017-07-05

**Authors:** Ali Pirsiavash, Ali Broumandan, Gérard Lachapelle

**Affiliations:** Position, Location and Navigation (PLAN) Group, Schulich School of Engineering, University of Calgary, Calgary, AB T2N 1N4, Canada; abrouman@ucalgary.ca (A.B.); lachapel@ucalgary.ca (G.L.)

**Keywords:** global navigation satellite systems (GNSS), signal quality monitoring (SQM), multipath detection, navigation reliability and integrity, signal processing

## Abstract

The performance of Signal Quality Monitoring (SQM) techniques under different multipath scenarios is analyzed. First, SQM variation profiles are investigated as critical requirements in evaluating the theoretical performance of SQM metrics. The sensitivity and effectiveness of SQM approaches for multipath detection and mitigation are then defined and analyzed by comparing SQM profiles and multipath error envelopes for different discriminators. Analytical discussions includes two discriminator strategies, namely narrow and high resolution correlator techniques for BPSK(1), and BOC(1,1) signaling schemes. Data analysis is also carried out for static and kinematic scenarios to validate the SQM profiles and examine SQM performance in actual multipath environments. Results show that although SQM is sensitive to medium and long-delay multipath, its effectiveness in mitigating these ranges of multipath errors varies based on tracking strategy and signaling scheme. For short-delay multipath scenarios, the multipath effect on pseudorange measurements remains mostly undetected due to the low sensitivity of SQM metrics.

## 1. Introduction

Many GNSS applications have strict reliability requirements as they involve Safety of Life (SoL) services and critical missions such as aviation, maritime and land transportation. In this context, the concept of reliability and service integrity is defined based on the level of trust in navigation solutions, given the variety of error sources affecting the system. One of the major phenomena that degrades the integrity of navigation solutions is multipath (e.g., [1]). Generally, GNSS signals received by a receiver are a combination of line-of-sight (LOS) and a number of non-line-of-sight (NLOS) components reflected off nearby obstacles one or more times before reaching the receiver antenna. This includes specular and diffuse reflections based on the characteristics of the reflector surface. Due to reflections, the received signals are different in power, delay, carrier phase and frequency, angle of arrival and polarization. The combination of these signals on the receiver antenna introduces multipath effects as widely discussed in the literature. Under multipath occurrence, a Delay-lock Loop (DLL) does not properly distinguish the actual peak of the correlation curve, as required for ideal code alignment. This results in a pseudorange error characterized by a significant multipath range error envelope (e.g., [2]). To overcome this effect, multipath mitigation techniques have been developed, resulting in well-known methods. A detailed discussion of these multipath countermeasure methods and their relative comparison can be found in [3]. As a general observation, some of these mitigating techniques attempt to minimize the effect of multipath by modifying the receiver tracking structure while others try to jointly estimate the multipath parameters and subsequently mitigate them. Polarization and spatial diversity (e.g., [4,5]) is another solution to reduce multipath which relies on different polarization and arrival angles of the reflected signals. These methods, however, require special hardware (antenna) considerations and modifications. Other solutions include three-dimensional building models (3DBMs) in urban environments (e.g., [6,7]) which also require additional geospatial information of nearby reflectors. However, in the context of reliability, the general approach to satisfy some level of navigation integrity is to detect and exclude (or de-weight) distorted measurements when exclusion does not degrade measurement geometry significantly (e.g., [7,8,9]). In this way, Signal Quality Monitoring (SQM) techniques have been developed to detect GNSS multipath distortions by incorporating monitoring correlators at the tracking level. References [10,11] incorporated different early-late correlators to define symmetric and asymmetric delta and ratio metrics to detect multipath distortions in the tracking correlation peaks. Reference [12] investigated an asymmetry test statistic called the Slope Asymmetry Metric (SAM) to evaluate signal quality in the presence of multipath. SAM compares the left and right slopes of the correlation curve and provides the receiver with timely alarms when one slope is steeper than the other as a result of multipath. References [13,14] exploited the Carrier-to-Noise-density ratio (C/N_0_) as a quality metric to weight GNSS measurements and improve positioning performance in multipath environments. Beside C/N_0_, [15,16] investigated a multipath detection approach using SAM and the DLL discriminator output to monitor the correlation peak quality. The correlator output samples are evaluated and integrity warning is set when the SQM metrics deviate from their nominal values. Based on a multi-correlator structure, [17] developed a signal quality index at the tracking level to evaluate the quality of GNSS signals and improve the performance of Receiver Autonomous Integrity Monitoring (RAIM) modules under multipath scenarios.

Despite the common use of SQM metrics, their sensitivity and effectiveness in detecting multipath have not been fully characterized. It has been shown that multipath affects SQM metric statistics (e.g., mean and variance) by distorting the correlation peak but there is no analytical discussion about how sensitive different SQM metrics are under different multipath scenarios. It has also been discussed that SQM-based monitoring results can be exploited for reducing the effect of multipath through de-weighting or excluding affected measurements but there are few investigations on how effective SQM metrics can be for multipath mitigation with respect to different tracking and signaling strategies.

This research carries out a characterization and performance evaluation of SQM techniques under different multipath scenarios. In Section 2, after modelling the received GNSS signals in the output of tracking correlators, monitoring correlators are defined based on their relative code delays from the reference tracking correlators. Different SQM metrics are defined in Section 3. Prior to setting an appropriate detection threshold, the statistical properties of the SQM metrics are investigated. The analytical discussion includes Binary Phase-shift keying (BPSK(1)) and Binary Offset Carrier (BOC(1,1)) modulations as the base schemes used for GPS and Galileo. For instance, BPSK(1) is the operational scheme used in GPS L1 C/A signaling. BOC(1,1) is also considered as the base scheme for Galileo and modernized GPS signals. Different implementations of BOC signaling (e.g., multiplexed BOC or MBOC) are also considered as the common baseline for Galileo and modernized GPS. In this context, analysis of the basic BPSK(1) and BOC(1,1) signals is of interest [18]. SQM metric variation profiles are proposed in Section 4 as a function of multipath relative delay and power. SQM metric effectiveness and sensitivity are then defined based on multipath range error envelopes and the proposed SQM profiles. These results are accompanied with an analytical discussion to evaluate SQM performance under different multipath scenarios. Two different correlator strategies, namely “Narrow Correlator” (NC) [19] and “High Resolution Correlator” (HRC) [20], are considered. These methods are commonly used in many receivers to mitigate multipath. Real data analysis is performed in Section 5 to validate the proposed SQM variation profiles and examine SQM detection performance for real static and kinematic multipath scenarios.

## 2. Signal Model

Received GNSS signals can be modeled as a combination of digitized signals corresponding to different PRNs as [21]:

(1) $$r(nT_{s})=\sum_{l=1}^{L}\sqrt{C_{l}}b_{l}(nT_{s}-\tau_{l})c_{l}(nT_{s}-\tau_{l})e^{j(2\pi (f_{IF}+f_{d,l})nT_{s}+\varphi_{l})}+\eta_{fe}(nT_{s})$$

where l is the PRN index; L is the number of satellites; $C_{l}$ is the power of the received signal from the lth satellite; $b_{l}$ is the binary navigation data; $c_{l}$ is the spreading code used to modulate the navigation data; $\tau_{l}$, $f_{d,l}$ and $\varphi_{l}$ are code delay, Doppler frequency and carrier phase introduced by the communication channel; $f_{IF}$ is the IF frequency and $f_{s}=1/T_{s}$ is sampling frequency. $\eta_{fe}(nT_{s})$ is front-end complex zero mean Gaussian noise. For each PRN, a reference tracking correlator multiplies the received signal by a corresponding PRN replica and the samples are integrated over a coherent integration time period. The output of the lth channel at the kth coherent integration epoch (time instant $kN_{s}T_{s}$) is given by:

(2) $$y_{l}[k]\equiv y_{l}(kN_{s}T_{s}^{})=\frac{1}{N_{s}}\sum_{n=(k-1)N}^{kN_{s}-1}r(nT_{s})c_{l}(nT_{s}-{\hat{\tau }}_{l})e^{-j(2\pi (f_{IF}+{\hat{f}}_{l})nT_{s}+{\hat{\varphi }}_{l})}$$

where $N_{s}$ is the number of samples in the coherent integration process. Using the sum of geometric series, Equation (2) can be rewritten as:

(3) $$y(kN_{s}T_{s}^{})=\sqrt{C}bR_{\tau }(\Delta \tau_{0})\frac{\sin(\pi \Delta f_{0}N_{s}T_{s})}{N_{s}\sin(\pi \Delta f_{0}T_{s})}e^{j\left(\pi \Delta f_{0}\left(\left(2k-1\right)N_{s}-1\right)T_{s}+\Delta \varphi_{0}^{}\right)}+\eta (kN_{s}T_{s}^{})$$

where the index l, which refers to the lth PRN, is omitted for simplicity. $\Delta \tau_{0}=\tau -\hat{\tau },$ $\Delta f_{0}=f_{d}-\hat{f}$ and $\Delta \varphi_{0}=\varphi -\hat{\varphi }$ are code, frequency and phase offsets between the received and the replica signal generated by the reference tracking correlator. $N_{s}T_{s}$ is the coherent integration time which is also noted by $T_{I}$. η consists of in-phase (I) and quadrature-phase (Q) noise and residual cross correlation terms approximated by a zero-mean Gaussian distribution with variance $\sigma_{0}^{2}=N_{0}/2T_{I}$ [22,23]. $N_{0}$ is the power spectral density of the receiver noise. $R_{\tau }(\cdot )$ is the correlation function which is related to the choice of the GNSS signaling scheme [10,24]. When tracking loops are locked and the received signal is stabilized in PLL mode, it is assumed that there are no tracking code and phase offsets and thus the in-phase output of the ith early or late correlator can be defined in the code-delay domain as follows:

(4) $$I_{c_{i}}^{}=\sqrt{C}R_{\tau }\left(c_{i}T_{c}\right)+\eta_{c_{i}}^{I}$$

where $T_{c}$ is the chip duration and $c_{i}T_{c}$ denotes the spacing of the ith early (for $c_{i}<0$) or late (for $c_{i}>0$) correlator from the reference prompt correlator. $\eta_{c_{i}}^{I}$ is the corresponding in-phase noise described above. The effect of binary data is neglected for the sake of simplicity. Since the tracking loops are locked and the received signal is tracked in PLL mode, the I branch is considered. If the receiver operates in a non-coherent mode, both I and Q branches should be taken into account [25].

## 3. SQM Metrics

Two types of correlators are considered in this section, namely the tracking and monitoring correlators, where the tracking correlators are used for tracking the signal and the monitoring correlators are used for signal quality monitoring. SQM metrics are defined as the combination of different tracking and monitoring correlators. The conventional Ratio and Delta SQM metrics are considered here as they make the base of most SQM metrics defined in the literature (e.g. [10,12,26,27,28]). The single sided ratio test (also known as asymmetric ratio or simply ratio test) is usually defined as the late correlator output divided by the prompt value. The reason for monitoring this ratio test is that the late side of the correlation curve is usually more distorted by typically delayed reflected signals [29]. The Delta metric (also known as symmetric ratio test) is a symmetric indicator which is designed to detect asymmetric correlation peaks based on the difference of either in-phase or absolute value of early minus late correlator outputs normalized by the prompt correlator [29]. The Double-Delta metric (as a specific case of SAM metric) is also defined here as the difference between two pairs of early-late correlators normalized by the prompt correlator.

Under nominal conditions, in low multipath open sky environments, calm ionospheric condition and the absence of other GNSS signal degradation errors, the output of each SQM metric is a random process whose statistics (e.g., mean and variance) is determined based on the location of the constituent correlators and receiver noise. References [10,24] investigated the nominal statistics of different SQM metrics for BPSK(1) and BOC(1,1) signaling schemes. The methodology is described in Appendix A. Table 1 summarizes the nominal mean and variance of the defined SQM metrics in which the tracking and monitoring early-late correlator spacings are 0.2 and 1 chips, respectively.

Note that the Double-Delta metric has been defined here based on the difference between monitoring and tracking pairs of early-late correlators. Under the alternate hypothesis, when multipath occurs, this definition of Double-Delta will result in similar performance as the Delta metric (defined based on the difference of monitoring correlators). This is because DLL keeps the tracking early-minus-late value around zero. However, under the null hypothesis (i.e., multipath free conditions), the difference of two pairs of early-late correlators alters the SQM metric variance based on the correlation between the tracking and monitoring correlators as shown in Table 1. This will affect detection performance and sensitivity whilst the number of monitoring correlators is the same as the Delta metric.

## 4. Characterization and Performance Analysis

The performance of the SQM metrics for multipath detection is now analyzed. The methodology is based on extracting SQM variation profiles and comparing them with the multipath tracking range error envelopes for different discriminators.

### 4.1. Multipath Rang Error Envelopes

**Figure 1:**
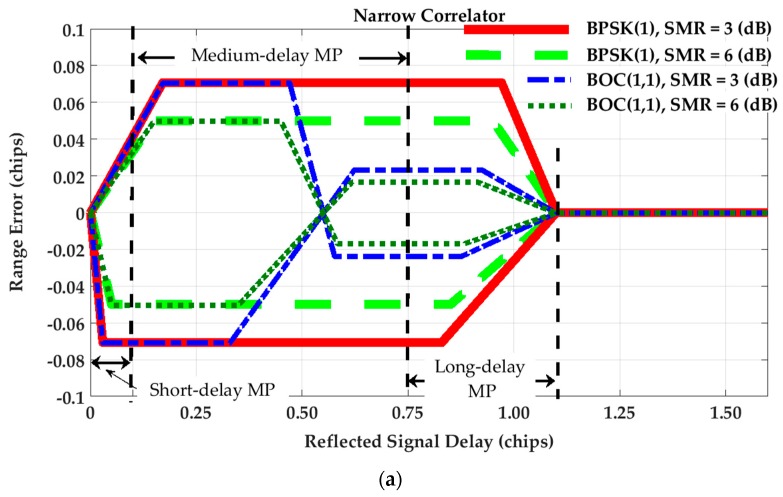

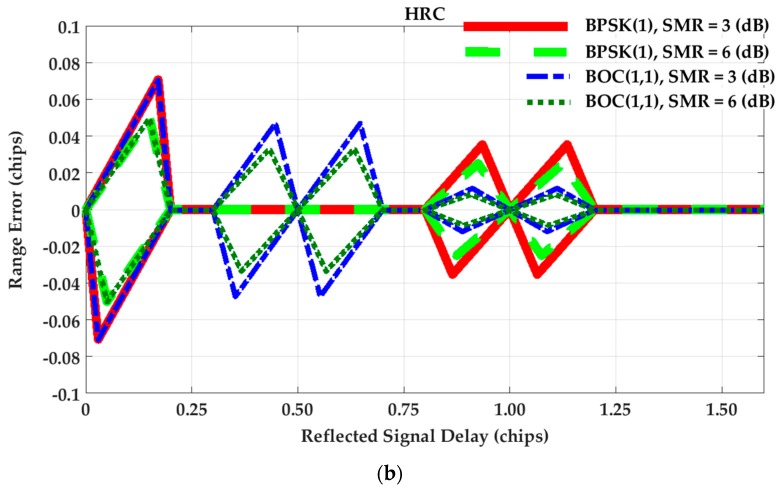
Multipath range error envelopes for (**a**) Narrow Correlator (NC) tracking strategy, BPSK(1) and BOC(1,1) signaling schemes; (**b**) High Resolution Correlator (HRC) tracking strategy, BPSK(1) and BOC(1,1) signaling schemes.

Figure 1 shows the multipath range error envelopes for Narrow Correlator (NC) and High Resolution Correlator (HRC) discriminators. The NC technique exploits narrow chip spacing between early and late tracking correlators (usually equal to or less than 0.2 chips) instead of using standard correlators with 1 chip spacing. This results in mitigating the effect of multipath on receiver tracking range error as shown in [19]. The HRC discriminator uses more than three correlators in the tracking loop [20]. The idea behind the HRC tracking strategy is to form a linear combination of correlators that results in a correlation function narrower than the standard one. Assuming a coherent tracking procedure, a DLL discriminator for NC and HRC techniques can be defined as follows [2,30]:

(5) $$D_{NC}=\left(I_{-d_{trk}/2}^{}-I_{+d_{trk}/2}^{}\right)$$

(6) $$D_{HRC}=\left(I_{-d_{trk}/2}^{}-I_{+d_{trk}/2}^{}\right)-0.5\left(I_{-d_{trk}}^{}-I_{+d_{trk}}^{}\right)$$

where $d_{trk}=0.2\;(\mathrm{chips})$ is considered to build up the tracking discriminator functions and extract the tracking range error envelopes for BPSK(1) and BOC(1,1) signaling schemes shown in Figure 1. To this end, a single reflection is considered and then the relative delay of the reflected signal (with respect to LOS signal) is swept through a range of values to evaluate DLL code misalignment and the consequent tracking range errors for in-phase and out-of-phase multipath components. These envelopes are shown in Figure 1 for 3 and 6 dB Signal-to-Multipath Ratio (SMR) values and for different tracking and signaling scenarios. Figure 1a also defines the approximate range of short, medium and long delay multipath considered here. Short-delay multipath is considered for reflected signal delays less than 0.1 chips (or about 30 m for the GPS L1 C/A case); medium-delay multipath is considered for the range of 0.1 to 0.75 chips and long-delay multipath covers the reflected signal delays longer than 0.75 chips.

According to Figure 1, the error envelopes are almost the same for all tracking strategies and signaling schemes for short-delay multipath. For path delays longer than approximately 0.5 chips, the corresponding tracking range error for the BOC(1,1) modulation is about one third of that of the BPSK(1) signal. This is because of the difference in the shape of correlation peaks for BPSK(1) and BOC(1,1). For the HRC technique, the maximum tracking range error is the same for both signals under the effect of short-delay multipath. Regarding medium-delay multipath, the BPSK(1) outperforms the BOC(1,1) signal as the resulting ranging error is reduced to zero. However, under long-delay multipath, the tracking range error of the BOC(1,1) is significantly lower than that of the BPSK(1) signal.

### 4.2. SQM Variation Profiles

**Figure 2:**
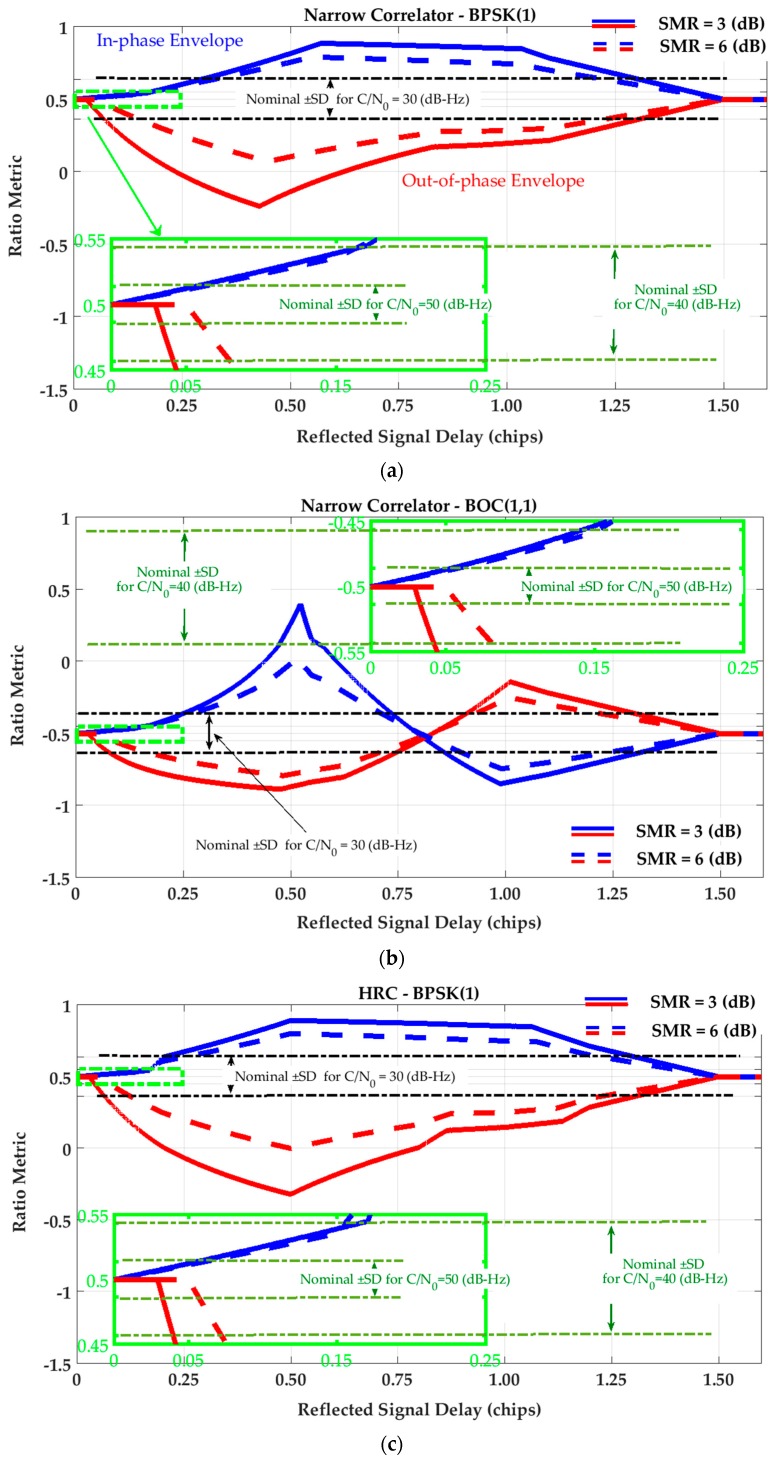

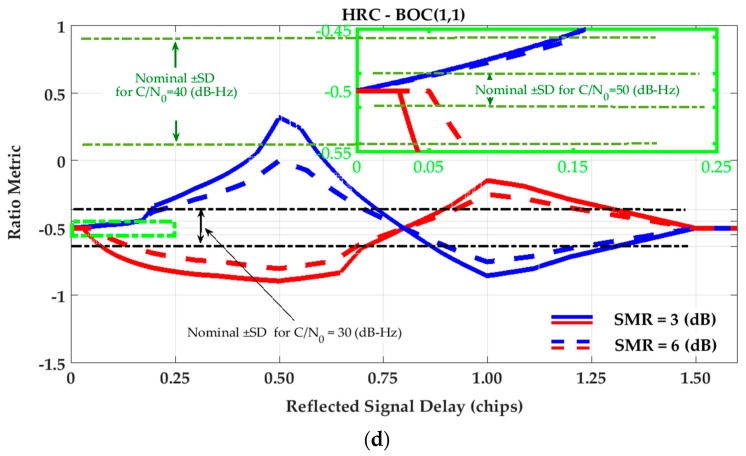
SQM variation envelopes of (single-sided) Ratio metric for (**a**) Narrow Correlator (NC) tracking strategy and BPSK(1) signaling; (**b**) Narrow Correlator (NC) tracking strategy and BOC(1,1) signaling; (**c**) High Resolution Correlator (HRC) tracking strategy and BPSK(1) signaling; (**d**) High Resolution Correlator (HRC) tracking strategy and BOC(1,1) signaling.

**Figure 3:**
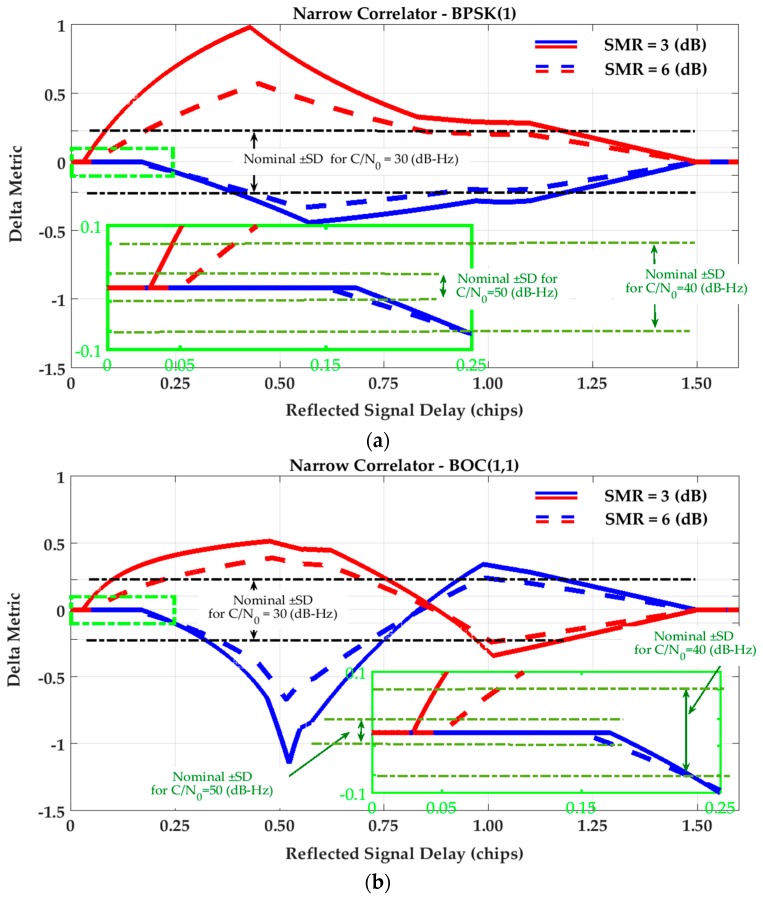

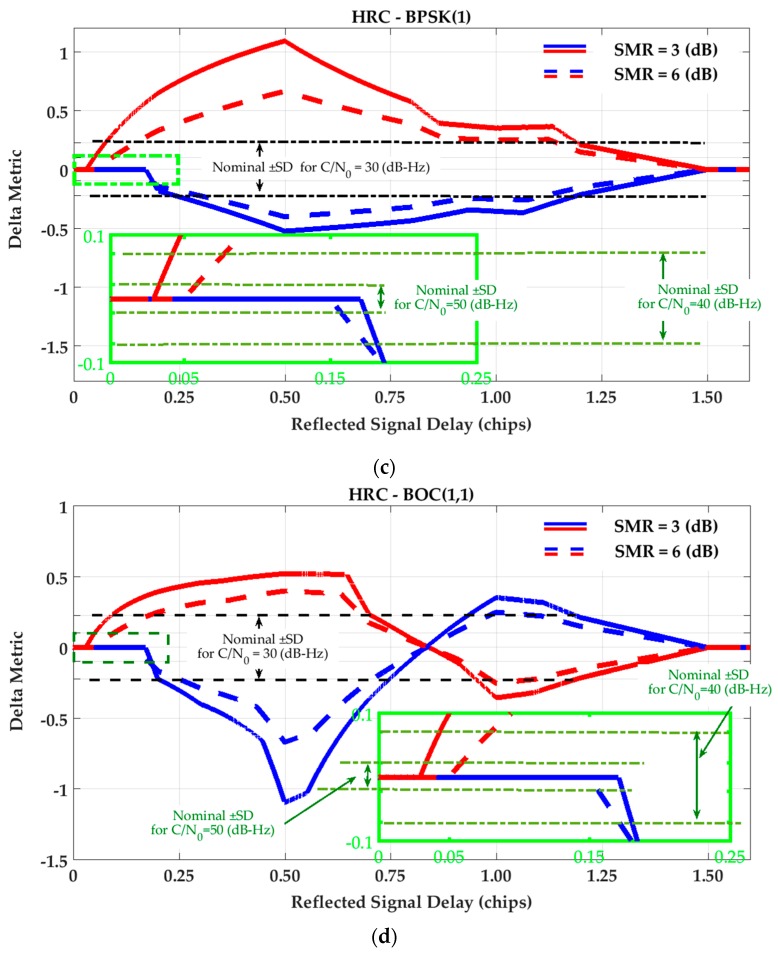
SQM variation envelopes of Delta metric for (**a**) Narrow Correlator (NC) tracking strategy and BPSK(1) signaling; (**b**) Narrow Correlator (NC) tracking strategy and BOC(1,1) signaling; (**c**) High Resolution Correlator (HRC) strategy and BPSK(1) signaling; (**d**) High Resolution Correlator (HRC) strategy and BOC(1,1) signaling.

Figure 2 and Figure 3 show the SQM variation profiles for Ratio and Delta metrics defined in Table 1. To extract these profiles, a single reflection is considered. Then the relative delay of the reflected signal is swept through a range of values to evaluate SQM metric outputs for in-phase (blue lines) and out-of-phase (red lines) multipath components for two signal-to-multipath ratio (SMR) values. It is observed that the SQM variation envelopes reach their maximum values around 0.5 chips where the late monitoring correlator is affected by the peak of the reflected correlation curve. Afterwards, whilst the multipath correlation curve passes the late monitoring correlator, the SQM variation envelopes decrease until multipath signal delay (τ) becomes greater than 1.5 chips and the multipath correlation curve no longer overlaps with tracking and monitoring correlators. For BOC(1,1) this reduction in variation envelopes shows a different behavior between 0.5 and 1 chips due to the different shape of the BOC(1,1) correlation curve.

In all cases, a lower SMR results in higher SQM variations due to the higher level of multipath relative power. Nominal Standard Deviations (SD) of the metrics are also shown in Figure 2 and Figure 3 for different C/N_0_ values. These values will now be used to evaluate the sensitivity of the SQM metrics in multipath detection. Based on a desired false alarm probability, a detection threshold is usually defined as a multiple of SD (for example three or five times) with clean data set. Therefore, for a given multipath scenario, if the SQM envelopes exceed the detection threshold, the SQM approach is considered sensitive, otherwise the multipath effect cannot be detected. As shown in the figures, for a given level of SMR, a higher C/N_0_ increases SQM sensitivity by reducing the nominal SD of the SQM metrics and thus lowering possible detection thresholds.

**Figure 4:**
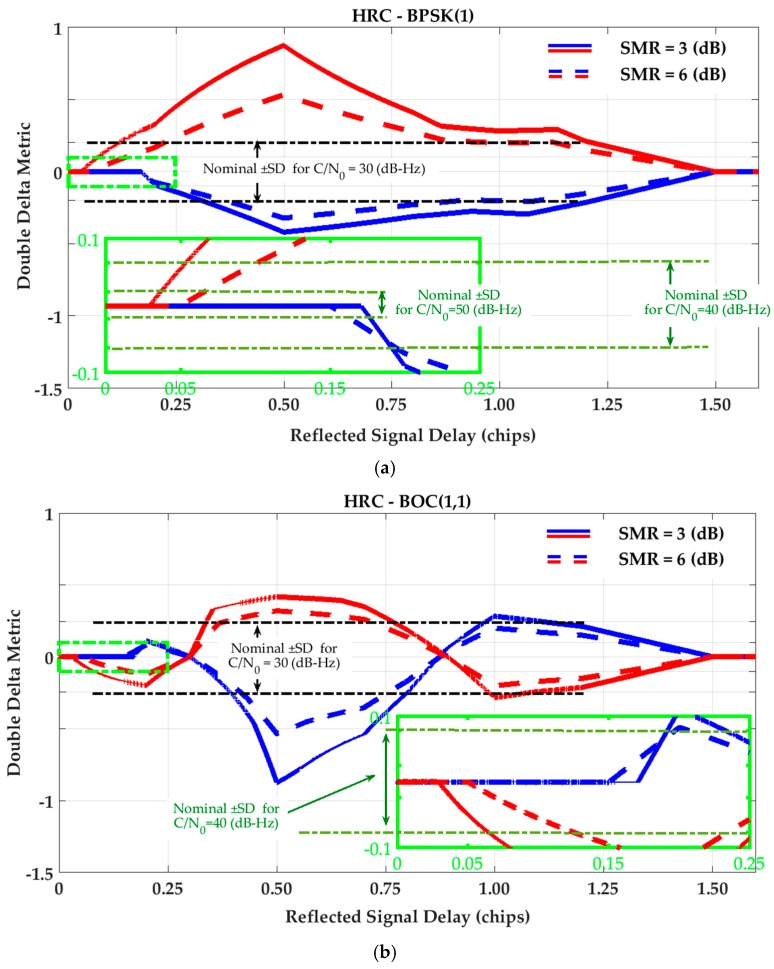
SQM variation envelopes of Double-Delta metric for (**a**) High Resolution Correlator (HRC) strategy and BPSK(1) signaling; (**b**) High Resolution Correlator (HRC) strategy and BOC(1,1) signaling.

For the Double-Delta SQM metric, the SQM envelopes are similar to those of the Delta metric (as the difference of monitoring correlators normalized by prompt) under the NC strategy. As discussed in Section 3, this is because the NC DLL discriminator keeps the tracking early-minus-late value around zero. However, under the HRC strategy, the corresponding envelopes are slightly different since HRC exploits different combinations of tracking correlators (see Equations (5) and (6)). Figure 4 shows the SQM variation envelopes for the Double-Delta metric for the HRC tracking strategy.

**Figure 5:**
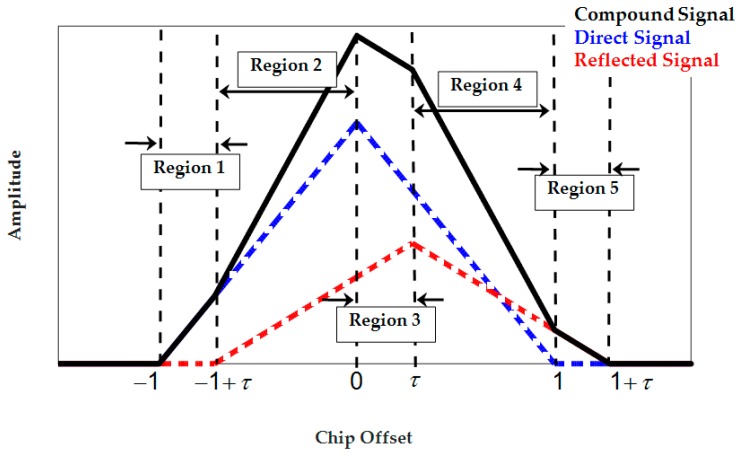
BPSK(1) correlation peak and its distortion under a single-ray multipath.

Inspection of the Delta and Double-Delta SQM envelopes in Figure 3 and Figure 4 shows that SQM outputs are zero (or below the nominal SDs) for some intervals of short-delay multipath. This multipath period induces range errors that are not detectable by SQM metrics. To elaborate on this phenomenon, consider Figure 5 which shows a BPSK(1) correlation function affected by multipath and divided into different regions. For BPSK(1) with the narrow correlator scheme, [31] showed that for short-delay multipath, the in-phase tracking range error envelope linearly increases from zero to $(1+\alpha )d_{trk}/2$ (α is the ratio of reflected and LOS signal amplitudes and $d_{trk}$ is the early-late tracking correlator spacing) with respect to the multipath signal delay (τ). Considering this, it can be shown that for $\tau \leq (1+\alpha )d_{trk}/2$, when $d_{trk}<d_{mon}\leq 1$ is hold ($d_{mon}$ is the monitoring correlator spacing), both early monitoring and tracking correlators are located in Region 2 and late tracking and monitoring correlators are both located in Region 4. Under these circumstances, since the correlators located in Regions 2 and 4 are affected by multipath symmetrically, the early-minus-late value for monitoring pairs of correlators will be equal to the tracking ones, resulting in zero output for the double Delta SQM metric. Even for the Delta metric, since DLL keeps the tracking early-minus-late value around zero, monitoring early-minus-late values will be kept around zero as well. For $(1+\alpha )d_{trk}/2<\tau$, depending on multipath delay and correlator spacing, the early correlators will mutually be in different regions or late correlators will be in different regions or early late correlators will be affected by the multipath correlation curve asymmetrically, resulting in non-zero SQM envelopes. This will occur until $(1+d_{mon}/2)<\tau$ when the multipath correlation curve has no overlap with tracking and monitoring correlators anymore. Using a similar argument, it can be shown that the out-of-phase envelope takes a zero value for $\tau \leq (1-\alpha )d_{mon}/2$. A similar argument can be made for the HRC discriminator and BOC(1,1) signaling schemes.

### 4.3. SQM Sensitivity and Effectiveness in Multipath Detection

To evaluate the performance and effectiveness of SQM approaches for multipath detection, the monitoring variation profiles should be compared with the multipath error envelopes. To this end, SQM “sensitivity” and “effectiveness” are now defined to make the subsequent discussion clearer.

The *sensitivity* of the SQM approach is defined based on the magnitude of the SQM variation profiles. If the SQM outputs exceed a given threshold, the multipath is detected and the SQM method is sensitive. Otherwise, the multipath signal is not detectable and the SQM metric is therefore not sensitive.

The *effectiveness* of the SQM approach is defined based on the magnitude of the multipath error. A SQM approach is effective when the SQM outputs are above the detection threshold (multipath is detected) and at the same time multipath has induced a significant error on range measurements. The effectiveness of a SQM approach is important since ideally SQM based measurement weighting should be based on its effectiveness and not sensitivity.

**Figure 6:**
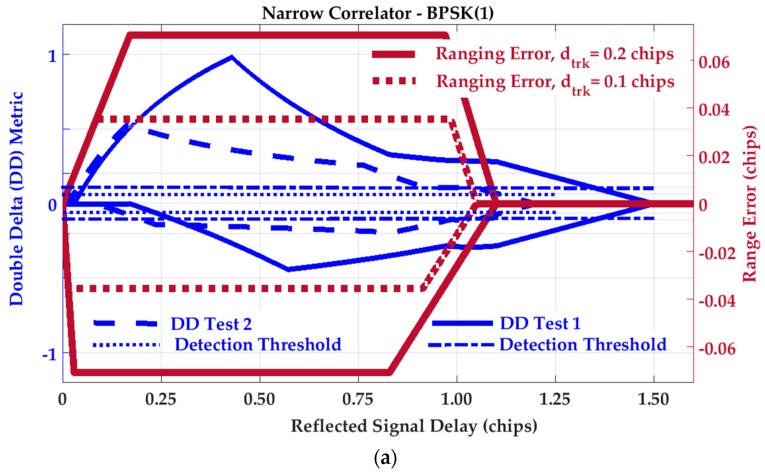

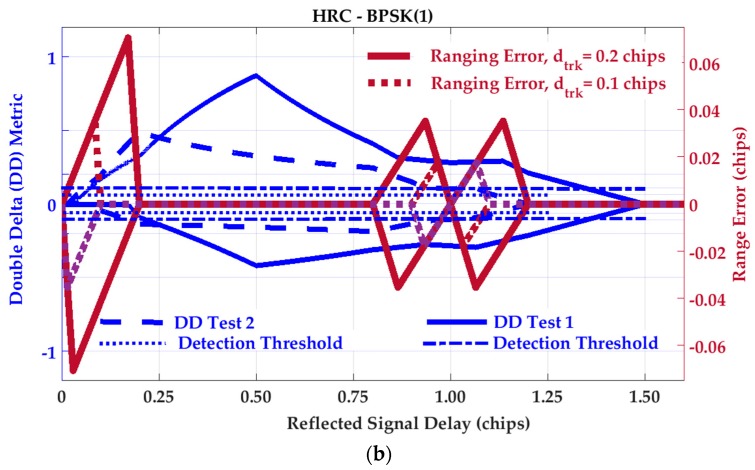
SQM profiles for Double-Delta metric and corresponding multipath error envelopes for (**a**) Narrow Correlator (NC) tracking strategy and BPSK(1) signaling scheme, SMR = 3 (dB); (**b**) HRC tracking strategy and BPSK(1) signaling scheme, SMR = 3 (dB).

Consider the Double-Delta (DD) metric for BPSK(1) signaling. To evaluate the effect of correlator spacing on the SQM effectiveness, two sets of correlator spacings are considered according to Table 2. In addition to the previously defined DD metric (Test 1), Test 2 is defined based on 0.1 and 0.4 chips tracking and monitoring the correlator spacing, respectively. The nominal statistics of Test 1 and Test 2 are summarized in Table 2. Figure 6 shows the SQM profiles of the Double-Delta Test 1 and Test 2 for SMR = 3 dB and BPSK(1) signaling (blue solid and dashed lines). For a better comparison, the corresponding ranging error envelopes are also plotted on the right-hand vertical axis in brown. For a C/N_0_ value of 45 dB-Hz, detection thresholds have been set as three times the corresponding nominal SD to satisfy a probability of false alarm of 0.0027.

According to Figure 6, for both NC and HRC discriminators, multipath with relative delays less than 10 m is not detectable. For the NC discriminator (Figure 6a), under medium and long-delay multipath, the deviations of the SQM metrics from the detection threshold (SQM sensitivity) coincide with the non-zero tracking range error envelopes (SQM effectiveness). For the HRC discriminator (Figure 6b), the Double-Delta SQM metric will not be effective for most medium-delay multipath scenarios when the resulting ranging errors decrease to zero. In order to evaluate the effect of correlator spacing, narrowing the tracking correlator mitigates the overall effect of multipath on tracking range errors (compare the solid and dotted brown lines in Figure 6) as is well known. In terms of SQM sensitivity, narrower monitoring correlators lower nominal SD and thus detection thresholds. This improves the sensitivity of the SQM metric in the case of short-delay multipath scenarios in which case narrower monitoring correlators results in higher SQM variations (compare the solid and dashed blue lines). In the case of the medium and long-delay multipath scenarios, both detection thresholds and SQM variation envelopes are decreased by narrowing the monitoring correlators.

With similar arguments applied to Figure 2, Figure 3 and Figure 4, the following conclusions can be made. For short-delay multipath, the effectiveness of the SQM metrics is almost the same for all tracking strategies and signaling schemes. In this range of multipath delays, the SQM detection outputs can be effective when the range error envelopes take non-zero values. In this area (especially for multipath delays less than 10 m) however, due to the low sensitivity of all the SQM metrics, it is possible that the resulting SQM values do not exceed the detection thresholds and thus multipath remains undetected for a realistic range of multipath power. For medium and long-delay multipath, the conclusions are as follows:

- Under NC discriminator and BPSK(1) signaling, the deviations of the SQM metrics from nominal values (SQM sensitivity) coincide with the non-zero tracking range error envelopes. This means that the SQM detection output can be exploited effectively for multipath mitigation by de-weighting (or excluding) distorted measurements (SQM effectiveness). This is true for all SQM metrics analyzed here.
- The effectiveness of the SQM metric for a NC discriminator with BOC(1,1) modulation is less than that of BPSK(1) (mostly for path delays longer than approximately 0.5 chips) where the corresponding ranging error is about one third of that of the BPSK(1) signal. In this scenario, according to the SQM profiles, the sensitivity of the metrics is also lower, which can be justified based on the different shapes of the correlation curves.
- In the case of the HRC discriminator, for BPSK(1) signaling, the SQM methods are not effective for medium-delay multipath due to negligible ranging errors. In this scenario, relying on SQM detection to de-weight or exclude measurements may even increase position errors due to geometry degradation. For BOC(1,1), the SQM can be effective for some intervals of medium-delay multipath when tracking range error envelopes take non-zero values. For long-delay multipath, comparing BPSK(1) and BOC(1,1) schemes, a lower SQM performance is observed for BOC(1,1) where the sensitivity of the SQM metrics is less and the multipath performance of the BOC(1,1) is significantly better than that of the BPSK(1).
- For a given SMR value, a higher C/N_0_ value increases SQM sensitivity by reducing the nominal variance of SQM metrics and lowering the detection threshold. In all cases, lower signal to multipath ratios (SMR) result in higher SQM sensitivity as expected.

### 4.4. Other Practical Issues

- The SQM variation envelopes show the maximum level of SQM sensitivity under specific multipath delay and power. However, in practice due to satellite motion and other effects, there are ever-present phase variations between LOS and multipath signals which cause SQM outputs to fluctuate between in-phase and out-of-phase envelopes. This will reduce the practical sensitivity of SQM metrics during multipath detection. The same argument applies to multipath range error envelopes and SQM effectiveness.
- In the extracted SQM profiles and multipath error envelopes, the power of reflected signals is assumed to be constant for different multipath delays. In practice, the reflected power should preferably be defined as a function of the multipath relative delay and a model of practical observations.
- In the signal monitoring approach, the practical statistics of SQM metrics may deviate from the nominal values based on the actual shape of the correlation function which depends on receiver parameters such as pre-correlation bandwidth and filter characteristics. In addition, the nominal statistics provided in Table 1 are calculated based on theoretical analysis with simplified approximations and assumptions. Therefore, for a reliable detection, the metric statistics and consequently detection thresholds should be calibrated based on practical observations.
- The SQM metrics variations are affected either by C/N_0_ value fluctuations or correlation peak distortions. Although the C/N_0_ can be also considered a monitoring metric itself, its effect on SQM-based detection performance is filtered through the tuning detection threshold based on the effective C/N_0_ at each epoch. If the effective C/N_0_ is smoothed, the effect of instant changes in C/N_0_ values will be projected on the SQM metric.
- The multipath detection process can be considered as a general maximizing procedure of the likelihood ratio by setting an appropriate detection threshold for each PRN. By setting this threshold, the SQM threshold excess can be monitored as a primary real-time indication of anomaly detection. However, to optimize detection performance and have more reliable detection (in terms of false alarm and detection probability), a filtering process should be applied at the expense of delayed decision-making. Herein, the detection procedure is defined based on a fixed interval detector called the ***M of N*** detection strategy usually used in GNSS acquisition procedures [32]. The ***M of N*** search detector takes a window of *N* samples and compares them to a predefined threshold. If *M* or more samples exceed the threshold, then the detection output will be 1 and otherwise 0. This procedure is repeated for the next window in the search pattern. With this detection strategy, the overall probability of false alarm in *N* trials is [32]:
  (7) $$P_{FA}=\sum_{n=M}^{N}\left(\begin{array}{c} N\\ n \end{array}\right)P_{fa}^{n}{\left(1-P_{fa}^{}\right)}^{N-n}=1-\sum_{n=0}^{M-1}\left(\begin{array}{c} N\\ n \end{array}\right)P_{fa}^{n}{\left(1-P_{fa}^{}\right)}^{N-n}$$
  where $\left(\begin{array}{c} N\\ n \end{array}\right)$ is the binomial coefficient of *n* and *N* integers referring to the number of combinations of *N* items taken *n* at a time. $P_{fa}^{}$ is the false alarm probability in each trial equals to 0.27% under a normal distribution assumption for the SQM metric and 3 times the standard deviation (±3σ) as the detection threshold.

## 5. Field Data Analysis

In this section, an actual data analysis is conducted for static and dynamic scenarios to validate the proposed SQM profiles and examine the sensitivity of the SQM approach in real multipath environments. These scenarios were carefully designed and considered to provide controlled conditions on the received signals affected by multipath corresponding to the discussion presented in Section 4.

### 5.1. Static Test Scenario

**Figure 7:**
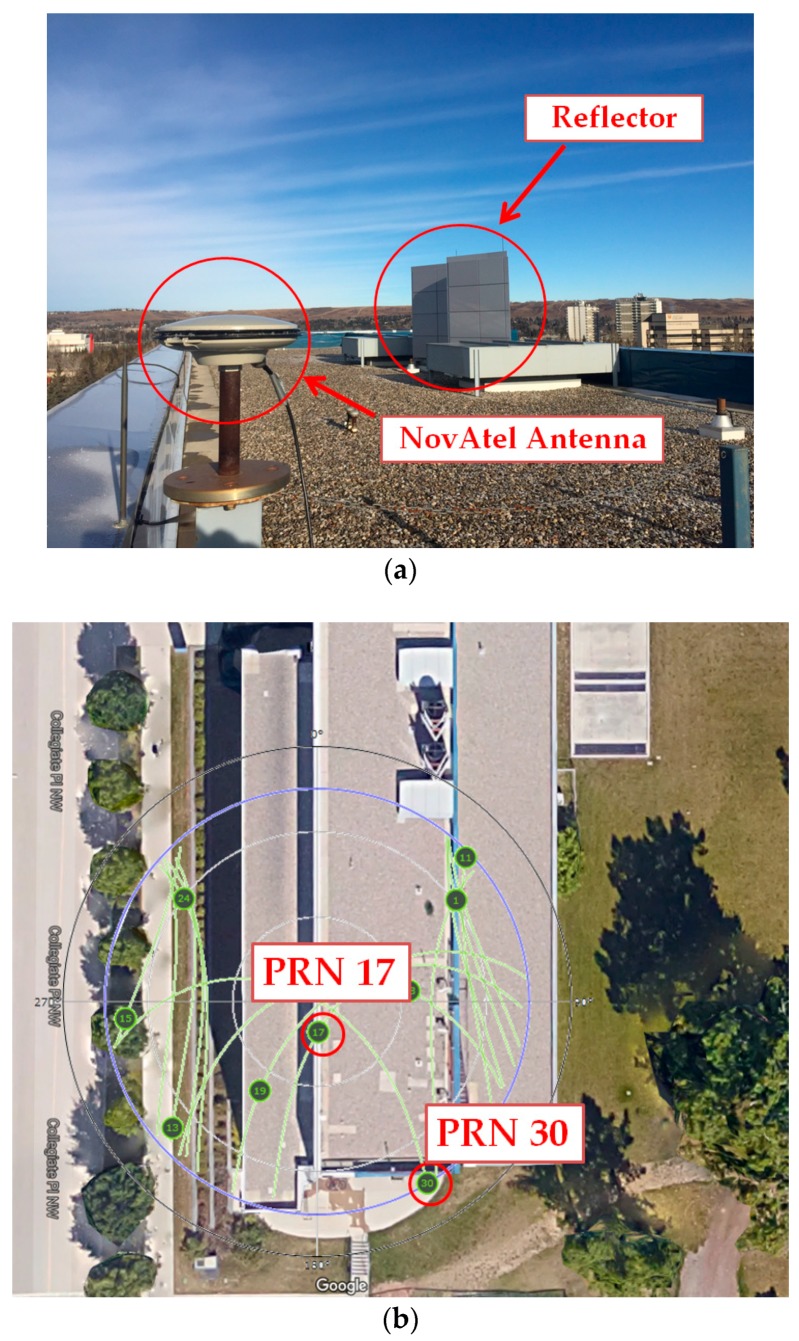
(**a**) Multipath data collection for static test scenario; (**b**) GPS satellites skyplot for static data collection.

GPS L1 C/A data was collected using a static rooftop antenna (NovAtel GPS-703-GGG) with one reflector in its vicinity to test a short-delay multipath scenario as shown in Figure 7a. In the first step, GPS data was collected for three days successively and analyzed by a PROPAK-V3 NovAtel receiver to monitor the effect of multipath on different PRNs during their full periods. This step was performed to find the appropriate data collection interval based on the repeatability of the multipath effect on a static antenna. In the next step, for the selected time interval, IF samples were collected at the same location for half an hour. The satellite skyplot is shown in Figure 7b for this interval. Some of the satellites, including PRN 17, have higher elevation angles and are expected to be less affected by multipath. PRN 30 has the lowest elevation and is more vulnerable to multipath. In this step, the data was collected using the same antenna (NovAtel GPS-703-GGG), down-converted and sampled using a NI (National Instruments) PXIe-1065 sampling front-end with a 10 MHz sampling frequency. The IF signals were then acquired and tracked in a software receiver providing correlator outputs to extract SQM results for different PRNs. The software receiver used is a modified version of [33] designed in Matlab. In this procedure, a narrow correlator discriminator with 0.2 chips early-late spacing was implemented. Monitoring correlators were located 1 chip apart; the coherent integration time was 20 ms.

**Figure 8:**
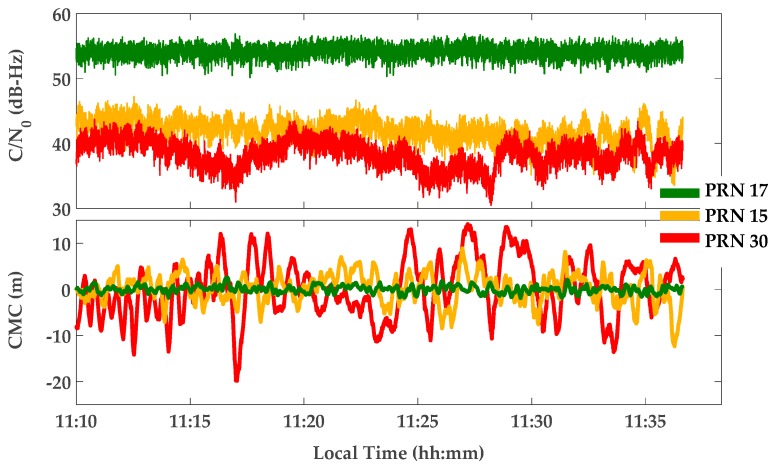
C/N_0_ and CMC measurements for three types of PRNs: PRN 17 with low multipath (clean PRN), PRN 15 with medium multipath and PRN 30 with high multipath.

Figure 8 shows Carrier-to-Noise-density ratio (C/N_0_) and Code-Minus-Carrier phase (CMC) measurements for a sample of PRNs. The C/N_0_ values are computed based on a classic technique known as the Narrowband-Wideband Power Ratio (NWPR) method [34]. CMC is a well-known technique to characterize and measure code multipath errors [35]. To this end, carrier phase measurements are subtracted from the corresponding pseudorange measurements. The results include code and carrier noise and multipath errors, carrier phase ambiguities and twice the ionospheric errors (due to ionospheric code delay and phase advance). The effects of ambiguity and ionosphere are estimated and removed using polynomial curve fitting. Neglecting the effect of carrier phase noise and multipath errors, the resulting value will be a measure of code multipath and noise.

**Figure 9:**
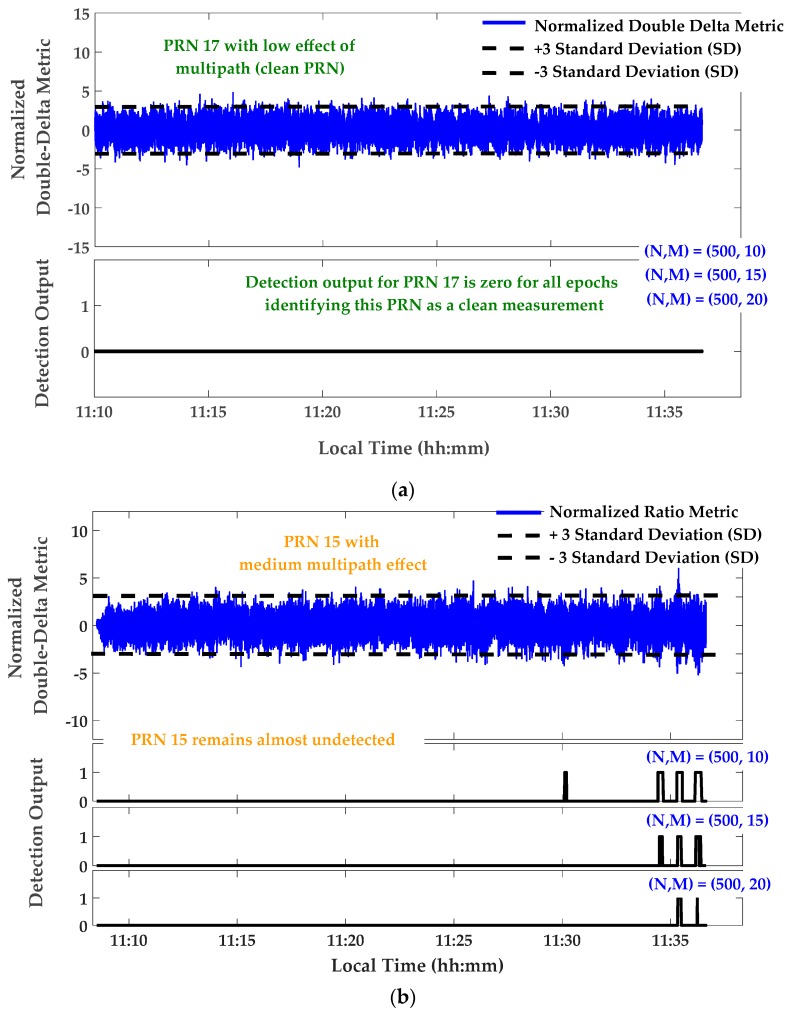

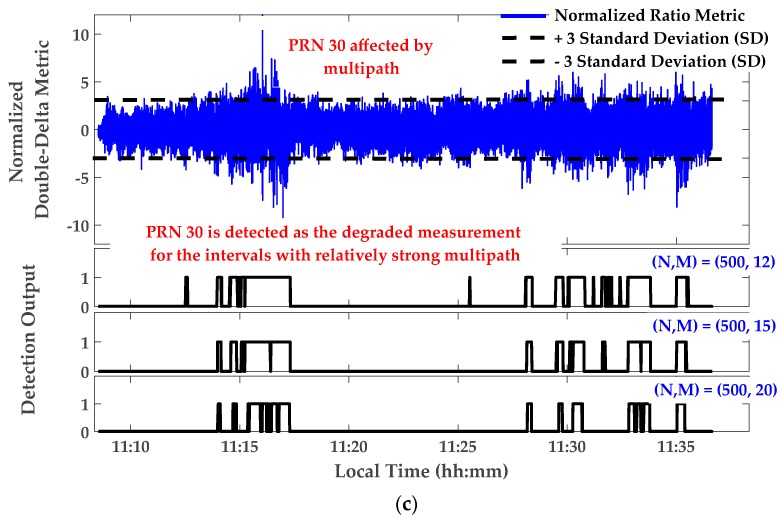
Signal quality monitoring—Normalized Double-Delta metric and corresponding detection outputs—Static scenario; (**a**) PRN 17; (**b**) PRN 15; (**c**) PRN 30.

Figure 9 shows monitoring results for the Double Delta metric defined in Table 1. Similar results, not presented here, were observed for Ratio and Delta metrics. In Figure 9, the Double-Delta metric outputs were calibrated and normalized using its theoretical variance according to Table 1. In this normalization, the C/N_0_ values were smoothed using a moving average window of 60 s. Afterwards, detection thresholds were fixed to ±3 times the Standard Deviation (SD) for the normalized metrics. The ***M of N*** detection strategy was used with *N* = 500 samples (or 10 s) and *M* = 12, 15 and 20, the overall probability of false alarm would be $2\times 10^{-8},\;1.6\times 10^{-11}\;\mathrm{and}\;1.8\times 10^{-14}$ respectively. As shown in Figure 9a, the output of the designed detection algorithm for relatively clean PRN 17 is zero for all epochs, identifying this PRN as a clean measurement. The detection output for PRN 15 and 30 are shown in Figure 9b,c, respectively. It is observed that, while PRN 15 with medium multipath (according to its CMC measurement) remains almost undetected, PRN 30 is detected as the affected measurement for the intervals with strong multipath. It is also observed that the aforementioned decision procedure detects multipath with a delay related to the length of the moving window in the detection strategy.

### 5.2. Kinematic Scenario

**Figure 10:**
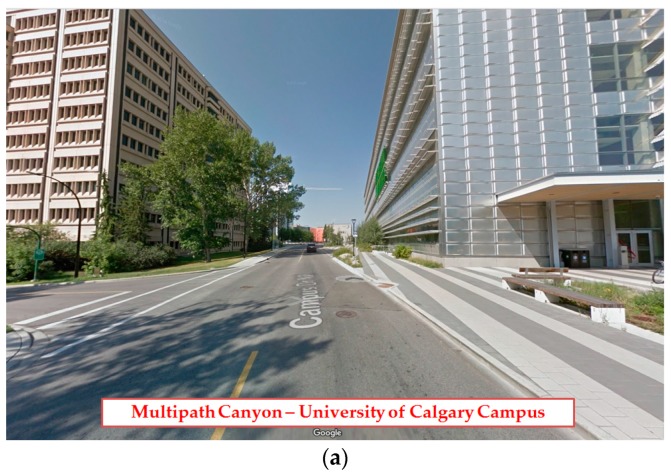

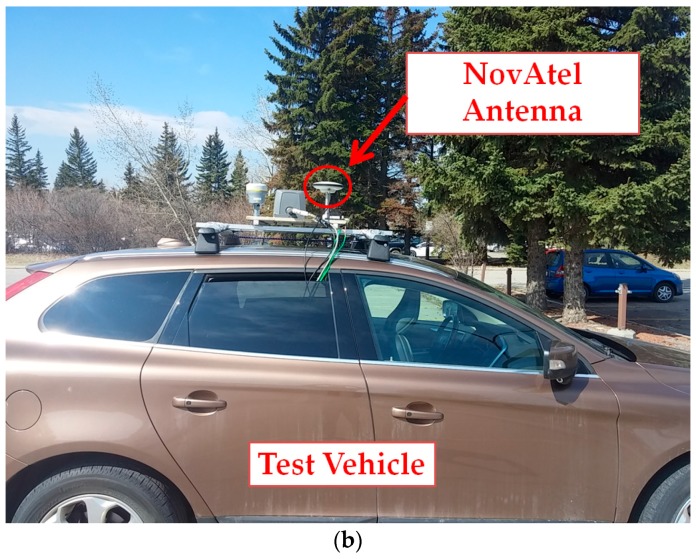
(**a**) Kinematic data collection—Multipath canyon—University of Calgary; (**b**) Test vehicle and NovAtel antenna for GPS data collection.

The sensitivity of the SQM approach for multipath detection was also examined in a kinematic scenario. Figure 10a shows the data collection environment. The building on the right has smooth surfaces which are considered as strong reflective sources. The GPS L1 data was collected with an antenna mounted on the test vehicle (Figure 10b) driving at a velocity of 1 m/s in the west direction through the multipath canyon shown in the figure. The data was down-converted and sampled using a Fraunhofer front-end with a 20 MHz sampling frequency. The GPS signals are then acquired and tracked in the software receiver (using a narrow correlator discriminator with 0.2 chips early-late correlator spacing) to monitor the signal quality as well as SQM metric outputs.

**Figure 11:**
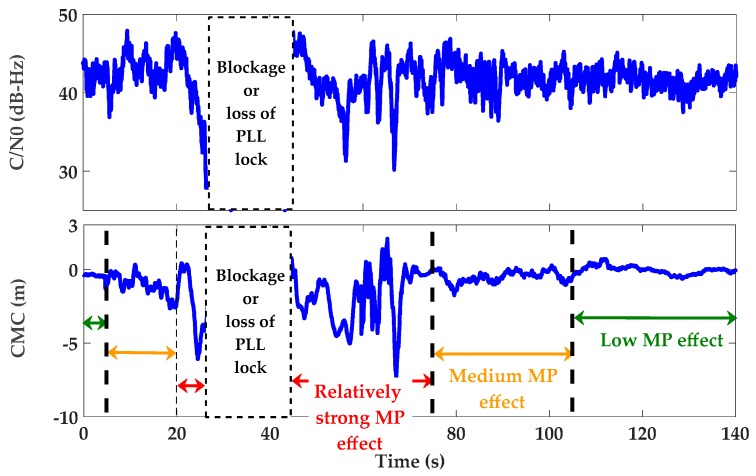
C/N_0_ and CMC measurements for PRN 21 (as a clear example of different levels of multipath during different segments of the trajectory).

**Figure 12:**
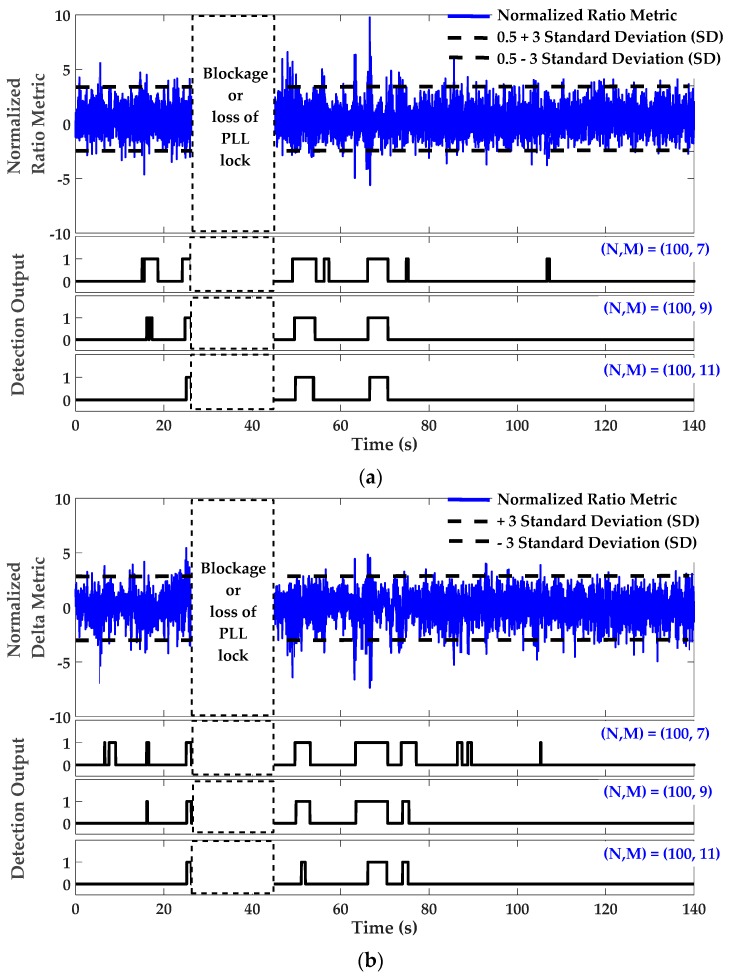

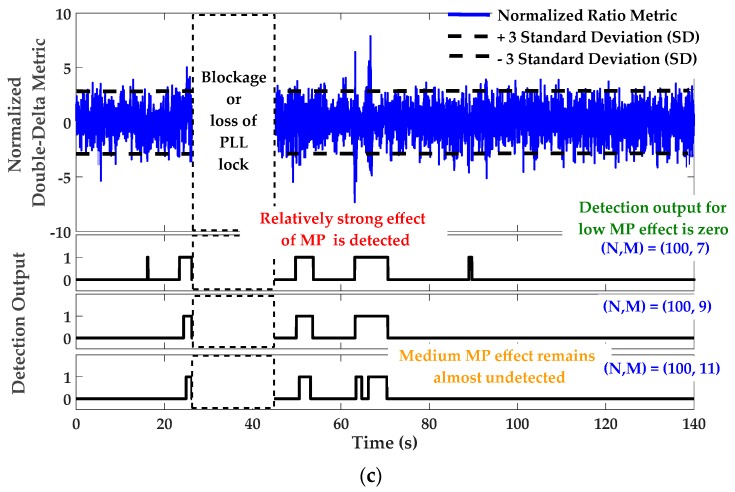
Signal quality monitoring and corresponding detection outputs for PRN 21—Dynamic scenario; (**a**) Normalized (single-sided or asymmetric) Ratio metric; (**b**) Normalized Delta metric; (**c**) Normalized Double-Delta metric.

Figure 11 and Figure 12 show the monitoring results of PRN 21 during the different segments of the trajectory. Similar results were observed for other PRNs. The kinematic data collection was started in a fairly open sky environment whose first 5 s is shown in the figures. The vehicle then passed through the multipath canyon for 100 s and ended in a fairly open sky environment (the last 35 s). The expected levels of multipath, corresponding to the vehicle trajectory, were observed in the resulting CMC measurements shown in Figure 11. In this plot, segments with relatively strong multipath are marked in red while medium and low multipath segments are marked in yellow and green, respectively.

Figure 12 shows the SQM and detection results for PRN 21. In addition to the Double-Delta metric, the monitoring results are shown for the Ratio and Delta metrics as defined in Table 1. The coherent integration time was 20 ms. All SQM metrics were calibrated and normalized using corresponding theoretical variances listed in Table 1. In this normalization, the C/N_0_ values were smoothed using a moving average window. Compared to the static scenario, the length of the moving average was reduced to 4 s due to the dynamic characteristic of the data and consequently faster multipath variations as a function of time. The detection thresholds were then fixed to ±3 times the normalized standard deviation (SD). The decision was based on the ***M of N*** detection strategy described above. Again, due to the dynamic data characteristics, the length of the sliding search window (*N*) was also reduced to 100 samples (2 s). According to Equation (7), *M* = 7, 9 and 11 were chosen and the overall false alarm probabilities are about $1.3\times 10^{-8},\;1.2\times 10^{-11}\;\mathrm{and}\;1\times 10^{-14}$, respectively. The detection output for the regions with low multipath (green region shown in Figure 11) is zero, indicating clean range measurement epochs for PRN 21. It is also shown that the medium effect of multipath (multipath with less than 3 m ranging error according to CMC measurement) is buried under SQM metric noise and remains almost undetected. However, for those epochs strongly affected by multipath (when multipath error is in the order of 5 m or more), the SQM metrics is sensitive and the detection output shows the occurrence of multipath.

## 6. Conclusions

This research investigated SQM variation profiles as critical requirements for the theoretical performance evaluation of the SQM approach for multipath detection. Ratio, Delta and Double-Delta metrics and different tracking and signaling schemes were investigated and different actual scenarios and comprehensive analyses were provided. Comparing the proposed profiles with the corresponding multipath error envelopes provides useful information to analyze the sensitivity and effectiveness of different SQM metrics for detecting multipath. The extracted results for the basic Ratio, Delta and Double-Delta metrics showed that although SQM is sensitive to medium and long-delay multipath, its effectiveness varies based on the tracking strategy and signaling scheme. Therefore, for a given scenario, effective utilization of SQM approach requires the joint analysis of both SQM sensitivity and effectiveness as proposed in this research. In short-delay multipath scenarios (especially for multipath delays less than 10 m), due to low sensitivity of SQM metrics, the multipath effect on the pseudorange measurements remains undetected. In this case, narrowing the monitoring correlators can improve the sensitivity of the SQM metrics. In all cases, a lower Signal-to-Multipath Ratio (SMR) results in higher SQM sensitivity and effectiveness, as expected. For a specific level of SMR, higher C/N_0_ increases SQM sensitivity by reducing the nominal variance of SQM metrics and lowering detection threshold. Actual measurements in static and kinematic multipath environments revealed the effectiveness of the proposed detection strategy. It was observed that a medium multipath error (e.g., multipath with less than 3 m ranging error) is buried under SQM metric noise and remains undetected. However, for those measurements strongly affected by multipath (for example when multipath error is in the order of 5 m or more), the SQM metrics are sensitive. Besides the aforementioned results, the underlying goal was to introduce the methodology of the SQM profiles in characterization and performance evaluation of SQM metrics defined for GNSS multipath detection.

## Figures and Tables

**Table 1:** Definition of SQM metrics and theoretical statistics.

SQM Metric	Definition	BPSK(1)		BOC(1,1)	
		Nominal Mean	Nominal Variance	Nominal Mean	Nominal Variance
(Single-sided or asymmetric) Ratio Metric	$m_{1}^{}=\frac{I_{+0.5}^{}}{I_{0}^{}}$	0.5	$\frac{0.75}{2\left(C/N_{0}\right)T_{I}}$	−0.5	$\frac{0.75}{2\left(C/N_{0}\right)T_{I}}$
Delta Metric	$m_{2}^{}=\frac{\left(I_{-0.5}^{}-I_{+0.5}^{}\right)}{I_{0}^{}}$	0	$\frac{2}{2\left(C/N_{0}\right)T_{I}}$	0	$\frac{2}{2\left(C/N_{0}\right)T_{I}}$
Double Delta Metric	$m_{3}^{}=\frac{\left(I_{-0.5}^{}-I_{+0.5}^{}\right)-\left(I_{-0.1}^{}-I_{+0.1}^{}\right)}{I_{0}^{}}$	0	$\frac{1.6}{2\left(C/N_{0}\right)T_{I}}$	0	$\frac{2.4}{2\left(C/N_{0}\right)T_{I}}$

**Table 2:** Double-Delta tests for BPSK(1) signaling.

SQM Metric	Metric Definition	Nominal Mean	Nominal SD	Nominal SD for C/N_0_ = 45 (dB-Hz)
Double Delta Test 1	$m_{3}^{}=\frac{\left(I_{-0.5}^{}-I_{+0.5}^{}\right)-\left(I_{-0.1}^{}-I_{+0.1}^{}\right)}{I_{0}^{}}$	0	$\sqrt{\frac{1.6}{2\left(C/N_{0}\right)T_{I}}}$	0.0356
Double Delta Test 2	$m_{4}^{}=\frac{\left(I_{-0.2}^{}-I_{+0.2}^{}\right)-\left(I_{-0.05}^{}-I_{+0.05}^{}\right)}{I_{0}^{}}$	0	$\sqrt{\frac{0.6}{2\left(C/N_{0}\right)T_{I}}}$	0.0218

## Appendix A

This section describes the methodology of analyzing statistics of the SQM metrics. Under nominal conditions, in low multipath open sky environments, calm ionospheric condition and the absence of other GNSS signal degradation errors, the output of each SQM metric is a random process whose statistics (e.g., mean and variance) is determined based on the location of the constituent correlators and receiver noise. Recall the in-phase output of the ith correlator in code-delay domain given by Equation (4) and repeated here for convenience:

$$I_{c_{i}}^{}=\sqrt{C}R_{\tau }\left(c_{i}T_{c}\right)+\eta_{c_{i}}^{I}$$

where $T_{c}$ is the chip duration and $c_{i}T_{c}$ denotes the spacing of the ith early or late correlator from the reference prompt correlator. C is the power of the received signal and $\eta_{c_{i}}^{I}$ is the corresponding in-phase zero-mean Gaussian noise whose variance is $\sigma_{0}^{2}=N_{0}/2T_{I}$. $T_{I}$ is the coherent integration time and $N_{0}$ is the power spectral density of the receiver noise. $R_{\tau }(\cdot )$ is the correlation function related to the choice of the GNSS signaling scheme [22]. With this definition, it can be shown that $I_{c_{i}}^{}$ is a random process with the following statistics [22].

(A1) $$\mu_{i}=E\left\{I_{c_{i}}^{}\right\}=\sqrt{C}R_{\tau }\left(c_{i}T_{c}\right)$$

(A2) $$\sigma_{i}^{2}=E\left\{{\left(I_{c_{i}}^{}-E\left\{I_{c_{i}}^{}\right\}\right)}^{2}\right\}=\sigma_{0}^{2}=\frac{N_{0}}{2T_{I}}$$

where E{⋅} denotes the statistical expectation. The covariance of the two $I_{c_{i}}^{}$ and $I_{c_{j}}^{}$ correlators is also calculated as follows [22]:

(A3) $$\sigma_{ij}^{}=\sigma_{ji}^{}=E\left\{\left(I_{c_{i}}^{}-E\left\{I_{c_{i}}^{}\right\}\right)\left(I_{c_{j}}^{}-E\left\{I_{c_{j}}^{}\right\}\right)\right\}=\sigma_{0}^{2}R_{\tau }\left(d_{ij}T_{c}\right)\;for\;\forall \;i\;and\;j$$

where $d_{ij}$ is the code distance (spacing) between the $I_{c_{i}}^{}$ and $I_{c_{j}}^{}$ correlators in the unit of chips. If the SQM metric *m* is considered as a general function of different correlators:

(A4) $$m=f(I_{c_{i}}^{},I_{c_{j}}^{},\ldots )$$

the variance of the metric can be calculated based on the error propagation law [10,24]:

(A5) $$\sigma_{m}^{2}=J\;D\;J_{}^{T}$$

where J is the Jacobian matrix containing the partial derivatives of the metric *m* with respect to the constituent correlators:

(A6) $$J=\left[\begin{array}{ccc} \frac{\partial m}{\partial I_{c_{i}}^{}} & \frac{\partial m}{\partial I_{c_{j}}^{}} & \cdots \end{array}\right]$$

D is the symmetrical covariance matrix of the correlators with the following structure:

(A7) $$D=\left[\begin{array}{ccc} \sigma_{i}^{2} & \sigma_{ij}^{} & \cdots \\ \sigma_{ji}^{} & \sigma_{j}^{2} & \cdots \\ \vdots & \vdots & \ddots \end{array}\right]=\sigma_{0}^{2}\left[\begin{array}{ccc} 1 & R_{\tau }(d_{ij}T_{c}) & \cdots \\ R_{\tau }(d_{ji}T_{c}) & 1 & \cdots \\ \vdots & \vdots & \ddots \end{array}\right]=\frac{N_{0}}{2T_{I}}\left[\begin{array}{ccc} 1 & R_{\tau }(d_{ij}T_{c}) & \cdots \\ R_{\tau }(d_{ij}T_{c}) & 1 & \cdots \\ \vdots & \vdots & \ddots \end{array}\right]$$

By defining the SQM metrics as a linear combination of different early and late correlators normalized by the prompt value, Equation (A5) can be rewritten as:

(A8) $$\sigma_{m}^{2}=J\;D\;J_{}^{T}=\left(\frac{1}{\sqrt{C}}\left[\begin{array}{ccc} g_{1} & g_{2} & \cdots \end{array}\right]\right)\left(\frac{N_{0}}{2T_{I}}\left[\begin{array}{ccc} 1 & R_{\tau }(d_{ij}T_{c}) & \cdots \\ R_{\tau }(d_{ij}T_{c}) & 1 & \cdots \\ \vdots & \vdots & \ddots \end{array}\right]\right)\left(\frac{1}{\sqrt{C}}\left[\begin{array}{c} g_{1}\\ g_{2}\\ \vdots \end{array}\right]\right)=\frac{h}{2\left(C/N_{0}\right)T_{I}}$$

where:

(A9) $$g_{a}=g_{a}\left(R_{\tau }\left(c_{i}T_{c}\right),\;R_{\tau }\left(c_{j}T_{c}\right),\;\ldots \right)\;for\;a=1,\;2,\;\ldots$$

(A10) $$h=h\left(R_{\tau }\left(c_{i}T_{c}\right),\;R_{\tau }\left(c_{j}T_{c}\right),\;R_{\tau }(d_{ij}T_{c}),\;\ldots \right)\;$$

are determined by the basic derivative calculations and matrix operations and based on the definition of the SQM metric, the correlator spacing of the constituent correlators and the shape of the correlation function. The nominal mean values of the SQM metrics can be approximated by simply replacing the prompt correlator with its mean value under nominal conditions.
